# Acetate fluxes in *Escherichia coli* are determined by the thermodynamic control of the Pta-AckA pathway

**DOI:** 10.1038/srep42135

**Published:** 2017-02-10

**Authors:** Brice Enjalbert, Pierre Millard, Mickael Dinclaux, Jean-Charles Portais, Fabien Létisse

**Affiliations:** 1 LISBP, Université de Toulouse, CNRS, INRA, INSA, Toulouse, France

## Abstract

*Escherichia coli* excretes acetate upon growth on fermentable sugars, but the regulation of this production remains elusive. Acetate excretion on excess glucose is thought to be an irreversible process. However, dynamic ^13^C-metabolic flux analysis revealed a strong bidirectional exchange of acetate between *E. coli* and its environment. The Pta-AckA pathway was found to be central for both flux directions, while alternative routes (Acs or PoxB) play virtually no role in glucose consumption. Kinetic modelling of the Pta-AckA pathway predicted that its flux is thermodynamically controlled by the extracellular acetate concentration *in vivo*. Experimental validations confirmed that acetate production can be reduced and even reversed depending solely on its extracellular concentration. Consistently, the Pta-AckA pathway can rapidly switch from acetate production to consumption. Contrary to current knowledge, *E. coli* is thus able to co-consume glucose and acetate under glucose excess. These metabolic capabilities were confirmed on other glycolytic substrates which support the growth of *E. coli* in the gut. These findings highlight the dual role of the Pta-AckA pathway in acetate production and consumption during growth on glycolytic substrates, uncover a novel regulatory mechanism that controls its flux *in vivo*, and significantly expand the metabolic capabilities of *E. coli*.

More than a century ago, Harden reported that the enterobacterium *Escherichia coli* excretes acetate when growing on excess fermentable sugars^1^. This phenomenon has been extensively investigated due to its physiological and applicative importance^2^,^3^,^4^,^5^,^6^,^7^. In *E. coli*, the main, constitutive, pathway of acetate production involves a combination of the phosphate acetyl-transferase (Pta) and acetate kinase (AckA). This way, acetyl-coA is converted into acetyl-phosphate then into acetate which is excreted^7^. Another route to form acetate is through oxidative decarboxylation of pyruvate by pyruvate oxidase PoxB^8^,^9^. *E. coli* is also able to consume acetate as a carbon and energy source to support growth. Acetate can be metabolized by two alternative pathways: the reversible Pta-AckA pathway (a low affinity route with a K_M_ for acetate of 7–10 mM)^2^,^10^, or the high affinity, irreversible acetyl-coA synthetase, Acs (with a K_M_ for acetate of 200 μM)^2^,^11^,^12^. Both pathways lead to the formation of acetyl-CoA (Fig. 1).

*E. coli* cells growing on excess glucose produce acetate but consume it only after the glucose is totally consumed^7^. This diauxic behavior is due to the catabolite repression exerted by glucose on acetate utilization. When glucose is in excess, the EIIA component of the phosphoenolpyruvate-carbohydrate phosphotransferase system PTS (the main glucose transport system in *E. coli*), mostly exists in its unphosphorylated form. This leads to the inhibition of adenylyl cyclase. Therefore cAMP levels are low and the transcriptional activator cAMP receptor protein (CRP), which is needed to transcribe *acs*, is inactive. The repression of *acs* expression prevents acetate consumption during the period of growth on glucose. In the absence of glucose, cAMP is produced and binds to CRP, which leads to *acs* expression and allows cells to consume acetate.

Consistent with the control of *acs* expression, simultaneous consumption of acetate and glucose is observed when catabolite repression is partially impaired^13^ or weakened^14^,^15^,^16^,^17^. In these conditions, *acs* is expressed and acetyl-CoA synthetase (Acs) is active, enabling acetate consumption to occur. The activity of Acs, in concert with the constitutive activity of Pta and AckA, results in setting up a metabolic cycle (Pta-AckA-Acs cycle) in which the acetate produced from glucose by Pta-AckA can be utilized by Acs^15^,^16^,^17^. This cycle leads to the simultaneous production and consumption of acetate.

Due to catabolite repression, the simultaneous consumption of glucose and acetate is normally expected not to occur. However, it was recently observed that acetate can be taken up and metabolized during exponential growth of wild-type *E. coli* K-12 strains on a mixture of glucose and acetate^18^. This observation was made in conditions of glucose excess. The ability of *E. coli* K-12 to consume acetate in such conditions is highly intriguing since *acs* is not expressed due to catabolite repression. This observation suggests acetate can still be utilized by another pathway upon glucose excess. Hence, it is likely that catabolite repression is not the unique determinant of acetate utilization in *E. coli*.

The first objective of this study was therefore to clarify the pathway by which acetate is consumed upon growth on excess glucose, i.e. in conditions of catabolite repression. The second objective was to identify the mechanism(s) that control(s) acetate metabolism in such conditions. To address these questions, we designed and carried out dynamic ^13^C-labeling experiments to quantify acetate production and consumption fluxes individually, and to identify the metabolic pathways supporting the fluxes. The results pointed out that the Pta-AckA pathway was responsible for both fluxes, and thermodynamic and kinetic *in silico* analyses suggested this pathway is thermodynamically controlled *in vivo* by extracellular acetate level. The proposed regulatory mechanism was validated experimentally on glucose and two other glycolytic substrates (gluconate and fucose). To evaluate the strength of the regulatory mechanism, experiments were carried out in conditions for which catabolite repression exerts its control on acetate metabolism, i.e. in batch cultures of the *E. coli* K-12 wild-type strain growing on excess glucose or on mixtures of glucose and acetate. The findings of this study have broad implications in our understanding of *E. coli* metabolism and its regulation.

## Results

### Acetate enters the TCA cycle during growth on glucose

We first verified that excretion and uptake of acetate actually occur simultaneously when *E. coli* K-12 MG1655 cells were grown on minimal medium supplemented with 15 mM glucose. Small amounts of uniformly ^13^C-labeled acetate were added to the cultures either in mid-exponential growth phase – during which acetate accumulated in the culture medium – or after glucose exhaustion, during which acetate is consumed. When ^13^C-acetate was added during the latter phase, incorporation of the ^13^C-label was observed in virtually all central carbon metabolites (Fig. 2). This includes not only intermediates of the TCA cycle, but also intermediates of carbohydrate pathways, indicating that acetate feeds both oxidative metabolism and gluconeogenesis in these conditions, as expected. When ^13^C-acetate was added during exponential growth on glucose, incorporation of ^13^C label was observed in TCA cycle intermediates, indicating assimilation of the exogenously supplied acetate. No ^13^C label was incorporated into intermediates of glycolysis, which is consistent with a low – if any – gluconeogenic activity in this condition. These results confirmed that extracellular acetate can enter into the cell and be metabolized even on excess glucose^18^. This utilization of acetate occurs while acetate was accumulating in the culture medium at the same time. In other words, the net accumulation of acetate in the medium upon glucose metabolism results from the balance between acetate production and acetate consumption, revealing the existence of a permanent and bidirectional exchange of acetate between the cells and the medium.

### Acetate is simultaneously produced and utilized via the Pta-AckA pathway

To evaluate the magnitude of the acetate exchange between cells and medium under excess glucose, we measured the separate unidirectional fluxes of acetate production and of acetate consumption. A dynamic ^13^C-labeling experiment was carried out by growing *E. coli* on minimal medium containing a binary mixture of 15 mM U-^13^C-glucose with 1 mM unlabeled acetate (Fig. 3a). While the concentration of uniformly labeled acetate (produced from glucose) increased along growth as a result of the production of acetate from glucose, the initial unlabeled pool decreased with time. A model was developed to simulate the labelling dynamics (Fig. 3b), in which the evolutions of the labeled and unlabeled pools of acetate were described as separate ODEs (see Methods section). The unidirectional fluxes of acetate production and acetate consumption were calculated by fitting the experimental data with the model. Acetate production and consumption were measured to be 7.7 ± 0.5 and 5.7 ± 0.5 mmol.g_DW_^−1^.h^−1^, respectively. The values of the unidirectional fluxes were three- to four-fold higher than the net acetate accumulation rate (2.2 mmol.g_DW_^−1^.h^−1^) and were of the same order of magnitude as the specific glucose consumption rate (Fig. 3c).

**Figure 1:**
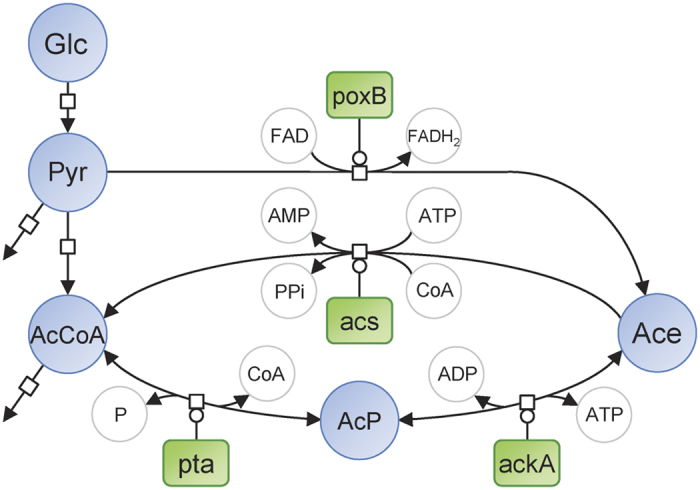
Representation of *E. coli* pathways involved in acetate metabolism, in Systems Biology Graphical Notation format (http://sbgn.org)^45^. Circles represent metabolites and rounded rectangles represent enzymes.

**Figure 2:**
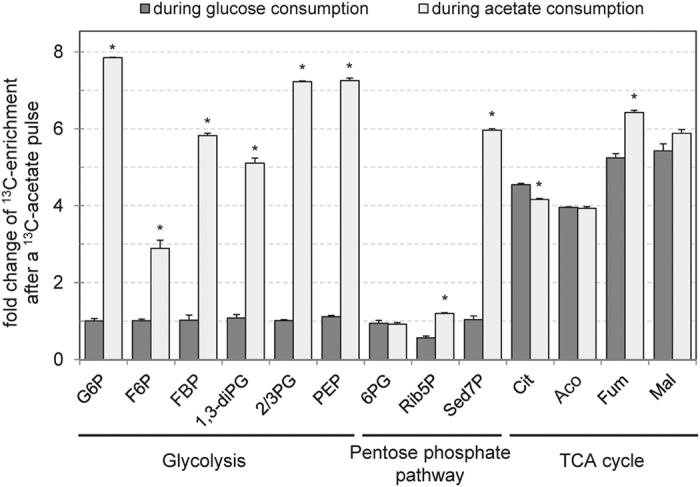
Incorporation of ^13^C atoms in central metabolites after a pulse of ^13^C-acetate during exponential growth on glucose or during the acetate consumption phase. *E. coli* K-12 MG1655 was grown on M9 supplemented with 15 mM glucose. ^13^C-acetate was added at a final concentration of 1 mM in mid exponential phase (dark grey bars) or during the phase of net acetate consumption (light grey bars). Intracellular metabolites were collected before each isotopic switch and one hour after the addition of labeled acetate, and the molecular ^13^C-enrichments of central metabolites were quantified by mass spectrometry. Results are displayed as the ratio between the ^13^C-enrichments of metabolite before and after the pulse. Asterisk (*) represents a p-value lower than 0.01 when compared to the label incorporation observed during glucose consumption.

**Figure 3:**
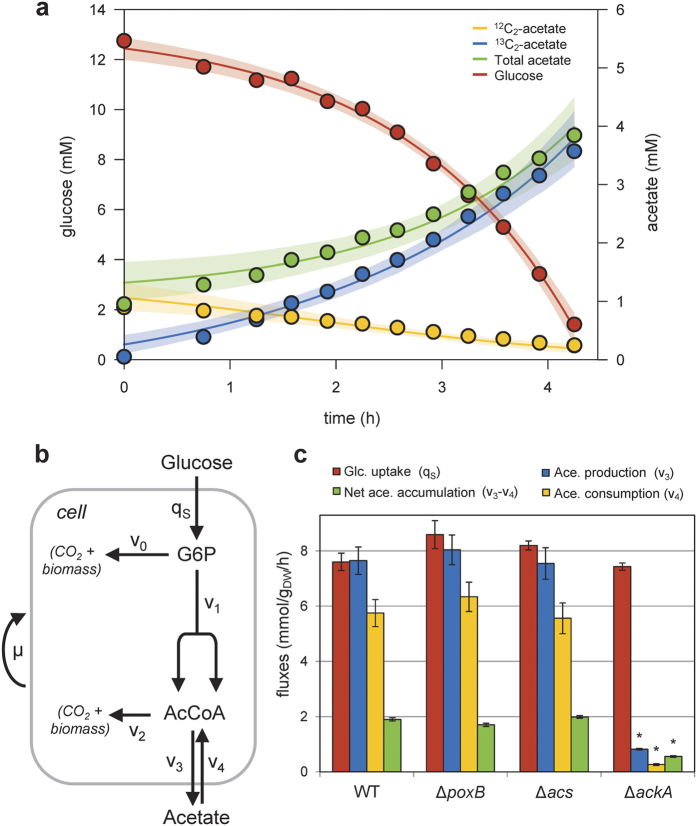
Quantification of acetate consumption and production fluxes by dynamic ^13^C-metabolic flux analysis. (**a**) Time-course profiles of glucose and total, labeled and unlabeled extracellular acetate concentrations. *E. coli* was grown on M9 supplemented with 15 mM ^13^C-glucose and 1 mM unlabeled acetate. The concentration of the four isotopomers of acetate was quantified by NMR every 30 minutes until the cells had consumed all the glucose (red circles). Unlabeled and 1,2-^13^C_2_-acetate are shown in yellow and blue, respectively. Concentrations of 1-^13^C_1_- and 2-^13^C_1_-acetate remained negligible (<0.1 mM) during the experiment. Total acetate concentration (in green) was calculated by summing the concentrations of all its isotopomers. Circles represent measurements, lines represent the best fit with their 95% confidence intervals (shaded areas). (**b**) Graphical representation of the model used to quantify acetate production and uptake fluxes. Details on this model are provided in the Methods section. (**c**) Glucose and acetate fluxes in *E. coli* K-12 MG1655 WT and its derivatives Δ*ackA*, Δ*acs* and Δ*poxB* strains. The experimental data shown in panel a were used to quantify the glucose uptake rate (red bars) and the net acetate accumulation flux (green bars) and its underlying production (blue bars) and consumption (orange bars) fluxes. Asterisk (*) represents a p-value lower than 0.01 when compared to the wild type strain.

The two pathways involved in acetate metabolism (*i.e*. the Pta-AckA and the Acs pathways) have different cofactor requirements (Fig. 1). The net cofactor balance of the complete acetyl-CoA/acetate cycle depends on the metabolic routes that are used for acetate production and acetate consumption, respectively. These routes can ultimately have an impact on the cellular energy balance. This is known to occur under glucose limitation, where acetate production via Pta-AckA and re-consumption via Acs lead to an energy-dissipating cycle, with a net balance of one ATP hydrolyzed into ADP per acetyl-coA recycled^17^. Assuming the simultaneous production and consumption of acetate observed in our study occurs entirely via the Pta-AckA-Acs cycle, the corresponding ATP expenditure would represent up to 5.7 mmol.g_DW_^−1^.h^−1^, *i.e*. 16% of the overall ATP needs of the cell^19^. To evaluate the actual impact of the simultaneous acetate production and consumption on cellular energetics, the contribution of each route to acetate production or consumption were quantified in strains deleted for enzymes from each pathway (Δ*poxB*, Δ*acs* and Δ*ackA*; Fig. 3c). The net acetate accumulation fluxes in the Δ*acs* and Δ*poxB* strains were similar to those of the wild-type. Consistently, the unidirectional fluxes of acetate production and acetate consumption were similar in the three strains. In contrast, the net accumulation of acetate in the Δ*ackA* strain was reduced by 71% compared to the wild-type. In this mutant, the unidirectional fluxes of acetate production and acetate consumption were reduced by about 90% compared to the wild-type. These data demonstrate that neither Acs nor PoxB play a role in acetate consumption in this condition while the Pta-AckA pathway can be responsible for both acetate production and acetate consumption. The latter pathway alone is sufficient to maintain a significant bidirectional flux of acetate. Contrary to the Pta-AckA-Acs cycle, this bidirectional process does not result in ATP wasting, and hence should have a minor impact on the ATP balance in *E. coli*.

### *E. coli* can consume acetate and glucose simultaneously

**Figure 4:**
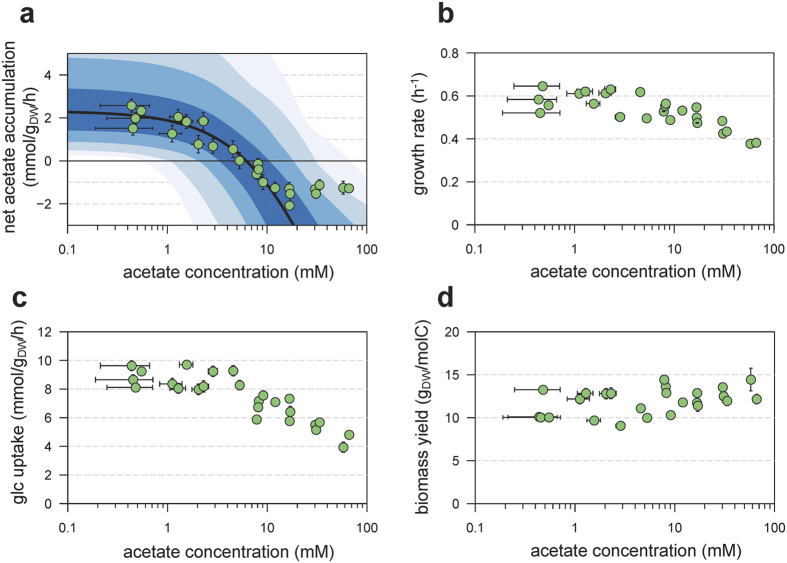
*In silico* analysis of the Pta-AckA pathway, and experimental validation of model predictions. The steady-state flux through the Pta-Ack pathway was simulated for different acetate levels (panel a, black line), with kinetic parameters taken from the literature and concentrations of ADP, ATP, CoA, acetyl-CoA and phosphate set to experimental values. To investigate the robustness of these predictions, sensitivity analysis was carried out by uniformly sampling all concentrations and parameters within ±10%, ±20%, ±30% and ±40% of their experimental values (dark blue to light blue, respectively). These predictions were compared to experimental values (green dots). Experimental growth rates (**b)**, glucose uptake rates (**c**) and growth yields (**d**) for different acetate levels.

The bidirectional flux of acetate, due to the reversibility of the Pta-AckA pathway, suggested that increasing extracellular acetate concentration could move the balance towards lower acetate production, and possibly towards net acetate consumption. If so, then extracellular concentration of acetate in the medium would decline. The free energy of the pathway (ΔG_Pta-AckA_) was calculated from the concentrations of relevant metabolites (see Methods). The sign of ΔG_Pta-AckA_ changed when acetate concentrations reached 6 mM, indicating that the net acetate flux could be reversed under the physiological range of metabolite concentrations. To get more quantitative insights into this question, a kinetic model of the Pta-AckA pathway was developed using parameters measured in this study or obtained from the literature (Fig. 4a; see Methods). This model was used to simulate the operation of the pathway for a wide range of acetate concentrations (from 10 μM to 100 mM). Consistent with experimental observations, the model predicted the accumulation of acetate in the medium at low acetate concentrations (below 10 mM). The Pta-AckA flux was predicted to decrease non-linearly when the acetate concentration increased, and then to reverse for acetate concentrations above 10 mM, resulting in net acetate consumption (Fig. 4a).

The predictions were tested experimentally by growing *E. coli* on minimal medium supplemented with glucose plus different concentrations of acetate (from 0.1 mM to 64 mM). Growth rate *μ*, specific glucose consumption rate *q*_*Glc*_, and specific acetate production rate *q*_*ac*_, were estimated for each condition (Fig. 4a–c). Growth rates and *q*_*Glc*_ were stable for acetate concentrations up to 8 mM (Fig. 4b and c), and then monotonously decreased as acetate concentration increased, consistently with previous reports^5^,^20^. In contrast, *q*_*ac*_ dropped as soon as the acetate extracellular concentration was above 1 mM (Fig. 4a). At an initial acetate concentration of 5.5 mM, *q*_*ac*_ reached zero meaning that acetate no longer accumulated in the culture medium. This is in excellent agreement with the value of 6 mM predicted by the kinetic model. Above the threshold concentration of 5.5 mM, *q*_*ac*_ was negative indicating that the extracellular concentration of acetate decreased in the culture medium. At acetate concentrations above 32 mM, acetate uptake was stabilized at 1.7 ± 0.3 mmol.g_DW_^−1^.h^−1^, which is lower than the predicted value (Fig. 4b). This may indicate saturation of acetate uptake or strong feedback inhibition by the rest of metabolism. These data also mean that glucose and acetate can be co-consumed by *E. coli*.

Exposure of *E. coli* cells to high acetate concentrations results in decreased growth rates while the growth yield *Y*_*X/S*_ remains constant (Fig. 4d). This confirms that the bidirectional operation of the Pta-AckA pathway does not massively dissipate energy otherwise one could expect a reduced growth yield.

### The Pta-AckA pathway supports acetate consumption in the presence of glucose

**Figure 5:**
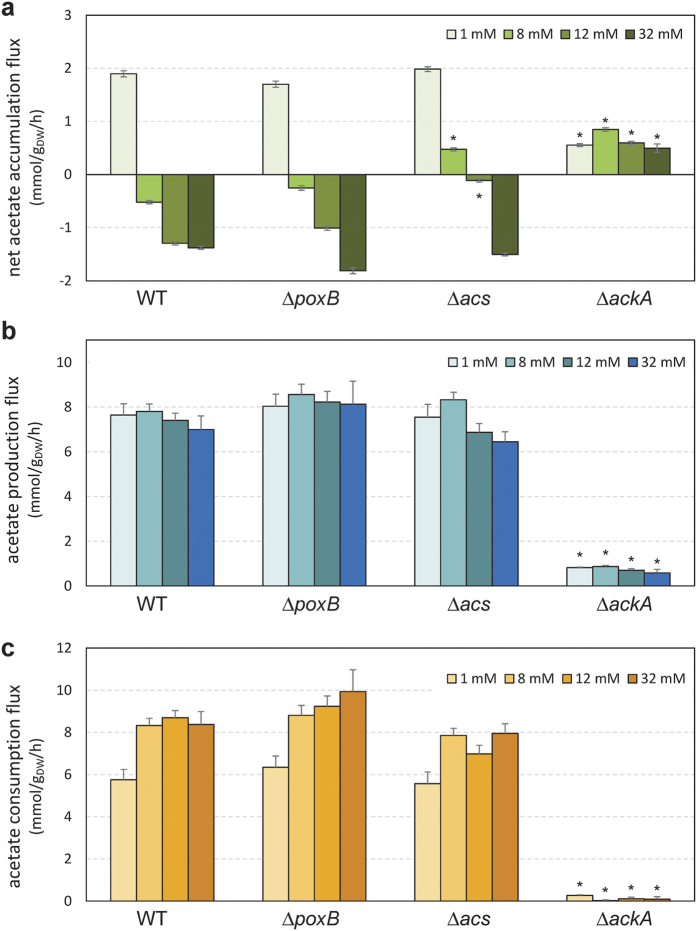
Net acetate accumulation flux and unidirectional production and consumption fluxes in *E. coli* K-12 MG1655 WT, Δ*ackA*, Δ*acs* and Δ*poxB* grown on glucose and different acetate concentrations. Dynamic ^13^C-metabolic flux analyses were carried out in which cells were grown in a medium containing uniformly ^13^C-labelled glucose +1, 8, 12 or 32 mM of unlabeled acetate. The fluxes of net acetate accumulation (**a**) and individual acetate production (**b**) and consumption (**c**) were quantified by fitting to experimental data, as detailed in the Methods. Asterisk (*) represents a p-value lower than 0.01 when compared to the wild type strain grown in the same condition.

To confirm that the Pta-AckA pathway is actually responsible for acetate consumption under excess glucose, the net flux of acetate accumulation, and the unidirectional fluxes of acetate production and acetate consumption were measured in the wild-type, Δ*acs*, Δ*poxB*, and Δ*ackA* strains for different acetate concentrations (Fig. 5). As observed above, the net accumulation of acetate in the wild-type strain reversed to a net consumption when acetate concentration was 8 mM or above (Fig. 5a). The behavior of the Δ*poxB* and Δ*acs* strains was similar to that of the wild-type, *i.e*. a shift from net accumulation to net consumption with increasing acetate concentrations. The net acetate accumulation rates were 1.7 ± 0.1 and 2.0 ± 0.1 mmol.g_DW_^−1^.h^-1^ at 1 mM of acetate and shift to −1.8 ± 0.1 and −1.5 ± 0.1 mmol.g_DW_^−1^.h^−1^ at 32 mM of acetate for Δ*acs* and Δ*poxB* strains respectively (Fig. 5a). In contrast, the *ackA* deletion virtually abolished both acetate production and consumption fluxes (Fig. 5b and c), under all tested acetate concentrations. A weak acetate accumulation (0.6 ± 0.2 mmol.g_DW_^−1^.h^−1^) was still observed in this mutant (Fig. 5a and b). This production can be due to the AckA independent transfer of phosphate by acetyl-P resulting in acetate formation^21^ or to the operation of biosynthetic pathways (methionine, cysteine, arginine, etc.) in which acetate is a metabolic by-product^17^. These results confirmed that, in the range of acetate concentrations tested, the Pta-AckA pathway is responsible for both the production and consumption of acetate, with no significant role of Acs and PoxB.

### The metabolic control of the Pta-AckA pathway ensures a rapid response of *E. coli* to changes in acetate availability

**Figure 6:**
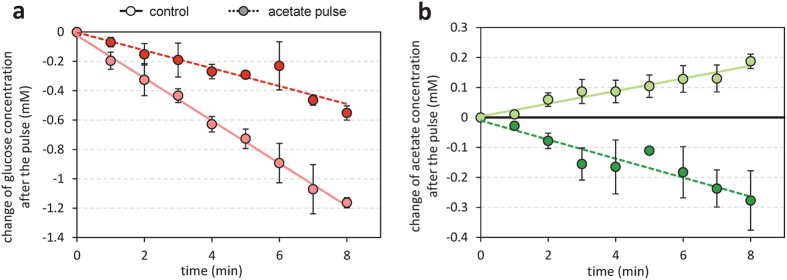
Short-term response of the Pta-AckA flux to an acetate pulse. *E. coli* was grown on M9 supplemented with 15 mM glucose. Changes in concentration of glucose (**a**) and acetate (**b**) were measured in mid-exponential growth phase with (dark green line) or without (control experiment, light green line) addition of acetate (at a final concentration of 30 mM) in mid-exponential growth phase.

An intuitive advantage of the proposed regulatory mechanism is that it could enable a rapid response of the Pta-AckA flux to changes in acetate availability. To evaluate the timing of this response, *E. coli* was grown on glucose, and in mid-exponential phase acetate was added to the culture to suddenly increase the acetate concentration from 3 to 30 mM. Changes in glucose and acetate concentrations were monitored every minute after the acetate pulse for 8 minutes and compared to a control experiment where no acetate was added (Fig. 6). The glucose consumption rate was reduced after the acetate pulse (Fig. 6a) which is consistent with the results obtained in steady-state conditions (Fig. 4b). Remarkably, while the acetate concentration increased in the control experiment, it decreased in the one or two minutes after the acetate pulse (Fig. 6b). Therefore, the addition of acetate provoked a rapid reversal of the net acetate flux from accumulation towards utilization. This fast response confirms the actual control of the Pta-AckA pathway *in vivo* by extracellular acetate levels and is consistent with the thermodynamic control of this pathway.

### Acetate is not only a by-product of glycolytic carbon sources but is also their potential co-substrate

**Figure 7:**
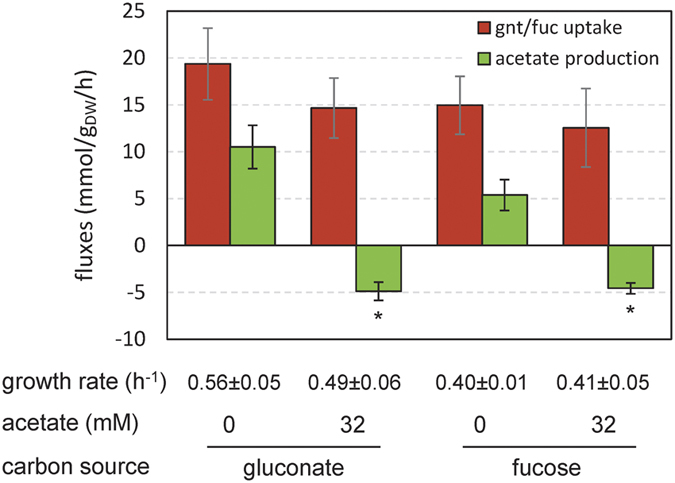
*E. coli* can co-consume acetate and glycolytic carbon sources. *E. coli* was grown on M9 supplemented with 15 mM gluconate or fucose. Growth rates, glycolytic substrate uptake rates and acetate accumulation rates were measured with or without acetate added initially to the medium (at a concentration of 32 mM). Asterisk (*) represents a p-value lower than 0.01 when compared to the same condition without acetate added initially to the medium.

We showed that glucose and acetate can be co-substrates for *E. coli* provided the latter compound is present in sufficiently high concentrations in the medium. We investigated whether this could also be the case for other glycolytic carbon sources, notably for some nutrients assumed to support growth of *E. coli* in the human gut. This was tested by growing *E. coli* on gluconate and fucose with or without addition of 32 mM acetate in the medium. As observed on glucose, acetate was produced from gluconate or fucose as sole carbon sources. When 32 mM acetate was added to the medium, acetate was utilized simultaneousy with the glycolytic substrates (Fig. 7). These observations extend the findings made on glucose and indicate that acetate is not only a by-product of glycolytic nutrients but can also be their co-substrate, depending solely on its extracellular concentration.

## Discussion

The data reported in this work show that *E. coli* can consume both acetate and glucose simultaneously upon exponential growth on excess glucose, in contrast to what is usually considered for this organism. This acetate consumption is not supported by acetyl-CoA synthetase. Not only is this enzyme repressed by catabolite repression in these conditions, but the simultaneous consumption of glucose and acetate is observed – to the same extent and even at very high acetate concentrations – in a mutant deleted for the *acs* gene. The potential contribution of PoxB to this phenomenon can also be eliminated. This acetate utilization is supported by the Pta-AckA pathway. Though known to be reversible, the latter pathway is considered to be involved only in acetate production. Our data show that the Pta-AckA pathway alone can support both the production and consumption of acetate upon excess glucose. Indeed, the pathway allows two opposite processes to occur concomitantly, including the conversion of acetyl-CoA into acetate and the reverse conversion of acetate into acetyl-CoA. The two unidirectional fluxes were measured and were significant. They were in the range of glucose uptake, and were 3–4 times higher than the net flux of acetate production or utilization. The overall result (acetate production or consumption) is the net balance between the two opposite processes. The net direction of the Pta-AckA pathway upon excess glucose, i.e. net production or net utilization of acetate, is controlled thermodynamically and is basically determined by the extracellular concentration of the compound. The experimental data and the kinetic model of the Pta-AckA pathway presented in this work are consistent and indicate that acetate is produced when its external concentration is below a threshold value of 8 mM. Above this threshold value, acetate is consumed.

The two main pathways of acetate metabolism in *E. coli*, i.e. the Pta-AckA and Acs pathways, appear to have very different mechanisms of control. Acs is controlled by catabolite repression, which is an active regulation process. This repression prevents the operation of the Pta-AckA-Acs cycle and hence avoids the spillage of energy. The Pta-AckA pathway is controlled thermodynamically – i.e. a passive process – which can have various benefits. First, the unidirectional fluxes of acetate production and acetate utilization are instantaneously adapted to the external concentration of acetate. This allows fine tuning of acetate metabolism according to its availability. Moreover, this regulatory mechanism allows metabolism to switch rapidly from acetate dissimilation to acetate assimilation during glycolytic growth, for instance in response to a sudden increase in acetate availability. Indeed, as shown by the acetate pulse experiments, the thermodynamic response time of the Pta-AckA pathway is of the order of 2 minutes, which is much faster than the time needed to functionally express new enzymes by hierarchical regulation (i.e. Acs). This ability to react fast is likely beneficial to face sudden changes in acetate concentration and might represent a competitive advantage in particular environments or conditions. This illustrates that metabolism is not self-contained in terms of control, but is highly sensitive to the environment. Finally, acetate assimilation via Acs can be seen as an effective but expensive way – due to the operation of the Pta-AckA-Acs cycle – to assimilate acetate in low or limited carbon environments whereas the Pta-AckA pathway is a low cost process to scavenge acetate in C-reach environments.

Acetyl-phosphate, the intermediate of the Pta-AckA pathway, is known to regulate many cellular processes in *E. coli* by phosphorylating or acetylating proteins and other molecules^7^,^22^,^23^. It is tempting to speculate that, due to the thermodynamic control of the Pta-AckA pathway, the intracellular level of acetyl-phosphate, hence its regulatory role, is modulated according to acetate availability. Indeed, our kinetic model predicts the accumulation of acetyl-phosphate when the extracellular concentration of acetate increases. The intracellular concentration in acetyl-phosphate is predicted to be 6 μM at 0.1 mM of acetate in the medium, and it increases to 3 mM at 60 mM acetate. These predicted concentrations are in good agreement with the intracellular levels of acetyl-phosphate reported in the literature^22^,^24^,^25^,^26^. The amplitude of the variation in acetyl-phosphate concentrations due to the thermodynamic control of the Pta-AckA pathway, as given by the model, is about 3 orders of magnitude. This is significant and consistent with the role of acetyl-phosphate as regulator, in particular in increasing the acetylation level of proteins *in vivo*^22^. Hence, the extracellular concentration of acetate might modulate the acetyl-phosphate pool through the thermodynamic control of the Pta-AckA pathway. This, in turn, would regulate many cellular processes, and likely result in fine tuning of metabolism according to acetate availability.

Our data indicate that acetate metabolism in *E. coli* is controlled to a large extent by the environment through the thermodynamic control of the Pta-AckA pathway. This control allows the bacterium to consume acetate even in conditions of glucose excess, provided the external concentration in acetate is high enough. This contrasts with the general consideration that acetate can be consumed only after exhaustion of glycolytic carbon sources. Our results indicate that the mode of utilization of glycolytic substrates and acetate by *E. coli* can be either sequential or concomitant depending on acetate availability. The net production of acetate by glucose-utilizing cells is abolished when the external concentration of acetate is 8 mM. Neither glucose uptake nor cell growth are altered in this condition, indicating that (net) acetate production is not an absolute requirement for *E. coli* to grow on glucose, be it to conserve energy^27^, or to control toxic organic acid pools^28^. In the laboratory, growth of *E. coli* on glucose is typically carried out in conditions resulting in low accumulation of acetate. In such conditions, the thermodynamics of the Pta-AckA pathway favors acetate production. The net production of acetate upon (exponential) growth on glucose can be the result of the thermodynamic driving force in the Pta-AckA pathway that drives acetate metabolism towards acetate production. In contrast to laboratory conditions, acetate concentration in the intestine is high (between 30 and 100 mM)^29^,^30^,^31^, well above the 8 mM threshold. From the thermodynamic point of view, such levels of acetate favor the net consumption of this compound in the intestine. It is therefore very likely that *E. coli* co-consumes acetate together with sugars in the intestine, in contrast to what is currently assumed^32^.

## Methods

### Bacterial strains

*Escherichia coli* K-12 MG1655 was selected as the model wild-type strain. BW25113 mutants^33^ were used to create their equivalent in the MG1655 background by bacteriophage P1-mediated transduction. Constructions were validated by PCR using internal, external and kanamycin-related primers (Supplementary Table S1). The list of strains is given in Supplementary Table S2.

### Cultures

*E. coli* was grown in M9 mineral medium complemented with 15 mM glucose^34^. Sodium acetate (prepared in solution at pH 7) was added to reach the required concentration. Acetate labelling assay was performed by adding 1.4 mM fully ^13^C-labeled acetate during mid-exponential phase or 2.4 mM one hour after glucose exhaustion. For ^13^C-labeling experiments designed for flux calculation, unlabeled glucose was replaced by uniformly ^13^C-labeled glucose (Eurisotop, France). Cultures were performed in duplicate or triplicate in shake-flasks at 37 °C and 200 rpm, in a volume of 200 mL. Growth was monitored by optical density at 600 nm using a Genesys 6 spectrophotometer (Thermo, USA), and a correlation factor of 0.37 g_DW_/L/OD unit was used to calculate biomass concentration.

### Extracellular metabolome analysis

Concentrations of labeled and unlabeled glucose and acetate were quantified in filtered broth (0.2 μm, Sartorius, Germany) by 1D ^1^H-NMR on a Bruker Ascend 800 MHz spectrometer equipped with a 5 mm QCI cryoprobe (Bruker, Germany), as detailed previously^35^.

### Intracellular metabolome analysis

Intracellular metabolites were extracted from cells exponentially-growing on glucose (without acetate initially present in the medium) using the differential sampling method. Adenine nucleotides (ATP and ADP) were measured by LC-MS and quantified using the Isotope Dilution Mass Spectrometry approach^36^. Absolute intracellular concentrations of ADP and ATP were calculated assuming a cytosolic volume of 1.77 × 10^−3^ L/g_DW_^37^.

For isotopic analyses, samples (500 μL) of culture broth were collected in 3 mL of quenching solution (20% H_2_O/40% methanol/40% acetonitrile) maintained at −20 °C^38^. Cellular extracts were dried, suspended in 200 μL water, and analyzed by mass spectrometry as described previously^39^. The ^13^C contents of intracellular metabolites were quantified from their isotopic patterns after correction for naturally abundant isotopes using the IsoCor software^40^. Statistical differences in labeling incorporation between the glucose consumption phase and the acetate consumption phase were calculated using Student’s paired t-tests with two tailed distributions.

### Dynamic ^13^C-metabolic flux analysis

To quantify acetate production and consumption fluxes, a dynamic model describing the propagation of ^13^C-atoms through the metabolic network was developed. This model includes seven reactions that represent glucose uptake, glycolysis, acetate production and consumption, and growth. Note that the system boundary considered here is the shake flask and not the cell. Therefore, cells accumulate at rate *X(t)·μ* (where *X(t*) denotes the concentration of biomass at time *t* and *μ* denotes the growth rate), glucose is consumed at rate *X(t)·qS* (where *qS* is the specific glucose uptake rate), and acetate accumulates at rate *X(t)·(v3*–*v4*) (where *v3* and *v4* are the specific acetate production and consumption rates, respectively). The dynamics of seven variables (concentrations of biomass and of labeled and unlabeled glucose, acetate and AcCoA) were simulated using the following system of ordinary differential equations (ODEs):


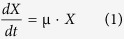



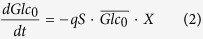



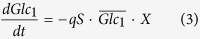



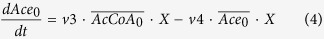



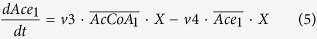



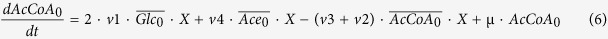



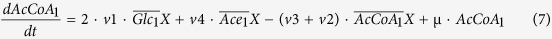


where the subscript *0* represents the unlabeled metabolite and *1* represents the ^13^C-metabolite.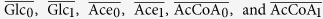 represent the abundances of the corresponding isotopic species relative to the total metabolite pool.

The system of ODEs defined by Eqs 1–7 was implemented in Fortran, and simulations were performed using the *lsoda* function of the *deSolve* package of R (v2.15, www.r-project.org), as detailed previously^41^. Free parameters (fluxes: *μ, qS, v3, v4*; and initial concentrations: Glc_0_, Glc_1_, Ace_0_, Ace_1_) were estimated using the *nlsic* algorithm developed by Sokol *et al*.^42^, by fitting the time-course concentrations of biomass, labeled glucose, and labeled and unlabeled acetate. Sensitivity analyses were carried out using the Monte-Carlo method (with 1,000 iterations). The relative standard deviations on the estimated parameters were below 10%. The scripts used to perform ^13^C-flux calculations are distributed in Supplementary Information under an open source license to ensure reproducibility and reusability.

Statistical differences in fluxes between the wild-type and mutant strains were calculated using Student’s paired t-tests with two tailed distributions.

### In silico analyses of the Pta-AckA pathway

Gibbs energy of the Pta-AckA pathway (Δ*G*_*Pta-AckA*_) was calculated for a wide range of acetate levels (10 μM to 100 mM) using the following equation:


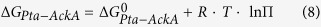


with


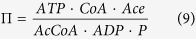


and


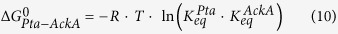


where *R* is the universal gas constant (*R* = 8.3143 J.K^–1^.mol^–1^), *T* is the temperature (*T* = 310 K), Π is the mass action ratio (calculated from reactant concentrations, eq. 9), and *K*_*eq*_ is the equilibrium constant of the reaction (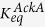 = 174 and 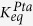 = 0.0281)^43^. Reactant concentrations were fixed at the values measured in this study (ATP = 2.4 ± 0.5 mM, ADP = 0.61 ± 0.18 mM) or elsewhere (P = 10 mM; CoA = 1.22 mM; AcCoA = 0.61 mM)^44^.

To investigate the impact of changes of acetate concentration on the flux through the Pta-AckA pathway, a kinetic model of this pathway was constructed. Rate laws and parameter values were taken from the literature^43^, and concentrations of phosphate, CoA, acetylCoA, ADP and ATP were set to their experimental values. The robustness of these simulations to parameter uncertainties was evaluated using a Monte-Carlo analysis (with 20,000 iterations), where all the concentrations and parameters were uniformly sampled within ±10%, ±20%, ±30% and ±40% of their experimental value. The R scripts used to perform these simulations and generate the figures are provided in Supplementary Information.

## Additional Information

**How to cite this article**: Enjalbert, B. *et al*. Acetate fluxes in *Escherichia coli* are determined by the thermodynamic control of the Pta-AckA pathway. *Sci. Rep.* **7**, 42135; doi: 10.1038/srep42135 (2017).

**Publisher's note:** Springer Nature remains neutral with regard to jurisdictional claims in published maps and institutional affiliations.

## Supplementary Material

Supplementary Tables

Supplementary Information
