# Supplementary material for: Acetate fluxes in Escherichia coli are determined by the thermodynamic control of the Pta-AckA pathway
Source: Sci Rep. 2017 Feb 10;7:42135. doi: 10.1038/srep42135 (PMC5301487; doi:10.1038/srep42135)
Supplement: Supplementary Tables [file srep42135-s1.pdf]

# Acetate fluxes in *Escherichia coli* are determined by the thermodynamic control of the Pta-AckA pathway

**Brice Enjalbert†, Pierre Millard†, Mickael Dinclaux, Jean-Charles Portais*, and Fabien Letisse**

LISBP, Université de Toulouse, CNRS, INRA, INSA, Toulouse, France

†these authors contributed equally to this manuscript

*portais@insa-toulouse.fr

# Supplementary Tables

**Supplementary Table S1.** Primers used in this work and their function.

| Name | Sequence (5' to 3') | Function |
|---|---|---|
| H1 acs | AACGCTTATGCCACATATTATTAACATCCTA CAAGGAGAACAAAAGCATG | External, 5' of the *acs* gene |
| H2 acs | GTTACCGACTCGCATCGGGCAATTGTGGGTT ACGATGGCATCGCGATAGC | External, 3' of the *acs* gene |
| 100 acs 5' | GGGAAAATTGACTGGCAGGA | Intern, toward 5' of the *acs* gene |
| 100 acs 3' | GGATCTTCGGCGTTCATCTC | Intern, toward 3' of the *acs* gene |
| H1 ackA | TGGCTCCCTGACGTTTTTTTAGCCACGTATC AATTATAGGTACTTCCATG | External, 5' of the *ackA* gene |
| H2 ackA | GCACCGCCAGCTGAGCTGGCGGTGTGAAAT CAGGCAGTCAGGCGGCTCGC | External, 3' of the *ackA* gene |
| 100 ackA 5' | TCTTCCACCTGCACGACACC | Intern, toward 5' of the *ackA* gene |
| 100 ackA 3' | TCGCTGGTCACTTCGGTCAG | Intern, toward 3' of the *ackA* gene |
| H1 poxB | GATGAACTAAACTTGTTACCGTTATCACAT TCAGGAGATGGAGAACCATG | External, 5' of the *poxB* gene |
| H2 poxB | CCTTATTATGACGGGAAATGCCACCCTTTT TACCTTAGCCAGTTTGTTTT | External, 3' of the *poxB* gene |
| 100 poxB 5' | TCGTGTTACCAGGCGACGTG | Intern, toward 5' of the *poxB* gene |
| 100 poxB 3' | CTTCTTCCGGCGTCACGACT | Intern, toward 3' of the *poxB* gene |
| K1 | CAGTCATAGCCGAATAGCCT | Intern, toward 5' of the kanamycine gene |
| K2 | CGGTGCCCTGAATGAACTGC | Intern, toward 3' of the kanamycine gene |

**Supplementary Table S2.** Strains used in this work.

| Name | Genotype | Origin |
|---|---|---|
| MG1655 | F- λ- ilvG- rfb-50 rph-1 | Blattner *et al*., 1997 |
| BW25113 Δ*acs* | rrnB3 ΔlacZ4787 hsdR514Δ(araBAD)567 Δ(rhaBAD)568 rph-1 Δacs::kan | Baba *et al*., 2006 |
| BW25113 Δ*ackA* | rrnB3 ΔlacZ4787 hsdR514Δ(araBAD)567 Δ(rhaBAD)568 rph-1 ΔackA::kan | Baba *et al*., 2006 |
| BW25113 Δ*poxB* | rrnB3 ΔlacZ4787 hsdR514Δ(araBAD)567 Δ(rhaBAD)568 rph-1 ΔpoxB::kan | Baba *et al*., 2006 |
| MG1655 Δ*acs* | F- λ- ilvG- rfb-50 rph-1 Δacs::kan | This work |
| MG1655 Δ*ackA* | F- λ- ilvG- rfb-50 rph-1 ΔackA::kan | This work |
| MG1655 Δ*poxB* | F- λ- ilvG- rfb-50 rph-1 ΔpoxB::kan | This work |
