# Supplementary material for: Acetate fluxes in Escherichia coli are determined by the thermodynamic control of the Pta-AckA pathway
Source: Sci Rep. 2017 Feb 10;7:42135. doi: 10.1038/srep42135 (PMC5301487; doi:10.1038/srep42135)
Supplement: Supplementary Information [file srep42135-s2.zip › Supplementary_Data/13C_flux_calculation/model_documentation.pdf]

# Dynamic isotopic model of *Escherichia coli* metabolism
# -
# Documentation

The dynamic model developed in this work describes the propagation of $^{13}$C-atoms through the metabolism *Escherichia coli* cultivated on glucose (batch experiments). It includes glucose uptake, biomass production, a simplified version of the glycolytic pathway, and acetate production and consumption reactions. The topology of the model is shown in Figure 1.

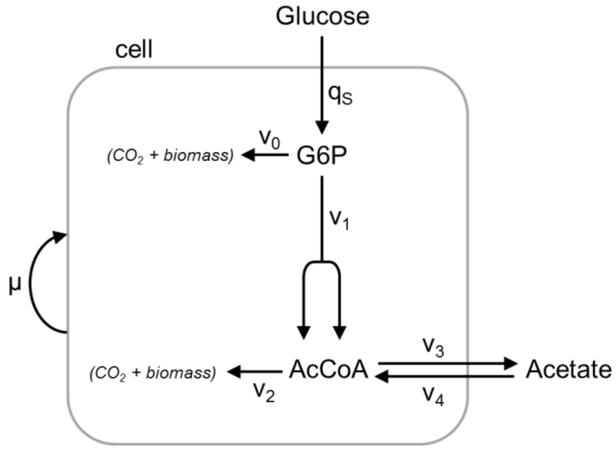


**Figure 1.** Representation of the model used to calculate fluxes.

*E. coli* is assumed to be at metabolic steady-state under the conditions investigated, i.e. concentrations of intracellular metabolites and intracellular fluxes are constant. At the intracellular level, glucose is consumed at rate $q_S$. According to the flux distribution measured under similar conditions by $^{13}$C-metabolic flux analysis [1], 70% of the glucose is directed towards AcCoA production via glycolysis (*v1*) and 30% is converted into $CO_2$ and biomass (*v0*). AcCoA fuels the TCA cycle via the citrate synthase (*v2*) and is converted into acetate via the phosphotransacetylase + acetate kinase and/or the pyruvate oxidase (*v3*). Finally, acetate can be converted back into AcCoA via the phosphotransacetylase + acetate kinase and/or the acetylcoA synthetase (*v4*).

It should be pointed out that the system boundary considered here is the shake flask and not the cell. Therefore, during a batch cultivation cells accumulate at the rate $\mu \cdot X$, where $\mu$ denotes the growth rate and $X$ denotes the concentration of biomass, and AcCoA accumulates at a rate $\mu \cdot AcCoA$. Finally, all the intracellular fluxes are multiplied by $X$, hence glucose is consumed at a rate $X \cdot qS$ and acetate accumulates at a rate equal to $(v3\text{-}v4) \cdot X$. All the reactions (except biomass synthesis) are considered separately for unlabeled and labeled metabolites.

This model contains a total of 7 variables (concentrations of biomass and of labeled and unlabeled glucose, acetate and AcCoA, Table 1) and 7 parameters (fluxes, Table 2). The system of ordinary differential equations is given hereafter (eq. 1-7).

(eq. 1) $\frac{dX}{dt} = \mu \cdot X$

(eq. 2) $\frac{dGlc_0}{dt} = -qS \cdot \frac{Glc_0}{Glc_0+Glc_1} \cdot X$

(eq. 3) $\frac{dGlc_1}{dt} = -qS \cdot \frac{Glc_1}{Glc_0+Glc_1} \cdot X$

(eq. 4) $\frac{dAce_0}{dt} = v3 \cdot \frac{AcCoA_0}{AcCoA_0+AcCoA_1} \cdot X - v4 \cdot \frac{Ace_0}{Ace_0+Ace_1} \cdot X$

(eq. 5) $\frac{dAce_1}{dt} = v3 \cdot \frac{AcCoA_1}{AcCoA_0+AcCoA_1} \cdot X - v4 \cdot \frac{Ace_1}{Ace_0+Ace_1} \cdot X$

(eq. 6) $\frac{dAcCoA_0}{dt} = 2 \cdot v1 \cdot \frac{Glc_0}{Glc_0+Glc_1} \cdot X + v4 \cdot \frac{Ace_0}{Ace_0+Ace_1} \cdot X - (v3+v2) \cdot \frac{AcCoA_0}{AcCoA_0+AcCoA_1} \cdot X + \mu \cdot (AcCoA_0 + AcCoA_1) \cdot \frac{AcCoA_0}{AcCoA_0+AcCoA_1}$

(eq. 7) $\frac{dAcCoA_1}{dt} = 2 \cdot v1 \frac{Glc_1}{Glc_0+Glc_1} \cdot X + v4 \cdot \frac{Ace_1}{Ace_0+Ace_1} \cdot X - (v3+v2) \cdot \frac{AcCoA_1}{AcCoA_0+AcCoA_1} \cdot X + \mu \cdot (AcCoA_0 + AcCoA_1) \cdot \frac{AcCoA_1}{AcCoA_0+AcCoA_1}$

| *Variable* | *Unit* | *Description* |
|---|---|---|
| X | $g_{DW}.L^{-1}$ | biomass concentration |
| $Glc_0$ | $mmol.L^{-1}$ | concentration of unlabeled glucose |
| $Glc_1$ | $mmol.L^{-1}$ | concentration of U-$^{13}$C-labeled glucose |
| $AcCoA_0$ | $mmol.L^{-1}$ | concentration of unlabeled acetylCoA |
| $AcCoA_1$ | $mmol.L^{-1}$ | concentration of U-$^{13}$C-labeled acetylCoA |
| $Ace_0$ | $mmol.L^{-1}$ | concentration of unlabeled acetate |
| $Ace_1$ | $mmol.L^{-1}$ | concentration of U-$^{13}$C-labeled acetate |

**Table 1.** Model variables. Initial values were estimated during parameter estimation, except for acetylCoA (total pool fixed at 10 $\mu mol.g_{DW}^{-1}$ and assumed to be at natural abundance at t=0).

| *Parameter* | *Unit* | *Description* | *Value* |
|---|---|---|---|
| qS | $mmol.g_{DW}^{-1}.h^{-1}$ | glucose uptake rate | Free parameter |
| mu | $h^{-1}$ | growth rate | Free parameter |
| v0 | $mmol.g_{DW}^{-1}.h^{-1}$ | output towards $CO_2$ and biomass | 30% of qS [1] |
| v1 | $mmol.g_{DW}^{-1}.h^{-1}$ | flux of acetylCoA production | 70% of qS [1] |
| v2 | $mmol.g_{DW}^{-1}.h^{-1}$ | output towards $CO_2$ and biomass | v4 - v3 - 2·v1 |
| v3 | $mmol.g_{DW}^{-1}.h^{-1}$ | flux from acetylCoA to acetate | Free parameter |
| v4 | $mmol.g_{DW}^{-1}.h^{-1}$ | flux from acetate to acetylCoA | Free parameter |

**Table 2.** Model parameters.

## References

1. Millard P, Massou S, Wittmann C, Portais JC, Letisse F (2014) Sampling of intracellular metabolites for stationary and non-stationary $^{13}C$ metabolic flux analysis in *Escherichia coli*. Anal Biochem 465C: 38-49.
